# Venezuelan Equine Encephalitis Virus Infection of Cotton Rats

**DOI:** 10.3201/eid1308.061157

**Published:** 2007-08

**Authors:** Anne-Sophie Carrara, Lark L. Coffey, Patricia V. Aguilar, Abelardo C. Moncayo, Amelia P.A. Travassos Da Rosa, Marcio R.T. Nunes, Robert B. Tesh, Scott C. Weaver

**Affiliations:** * University of Texas Medical Branch, Galveston, Texas, USA; † Evandro Chagas Institute, Belém, Brazil

**Keywords:** Encephalitis, alphavirus, reservoir host, cotton rat, arbovirus, research

## Abstract

Venezuelan equine encephalitis virus (VEEV) is an emerging pathogen of equids and humans, but infection of its rodent reservoir hosts has received little study. To determine whether responses to infection vary among geographic populations, we inoculated 3 populations of cotton rats with 2 enzootic VEEV strains (Co97-0054 [enzootic ID subtype] and 68U201 [enzootic IE subtype]). The 3 populations were offspring from wild-caught cotton rats collected in a VEE-enzootic area of south Florida, USA; wild-caught cotton rats from a non–VEE-enzootic area of Texas, USA; and commercially available (Harlan) colony-reared cotton rats from a non–VEE-enzootic region. Although each population had similar early viremia titers, no detectable disease developed in the VEE-sympatric Florida animals, but severe disease and death affected the Texas and Harlan animals. Our findings suggest that the geographic origins of cotton rats are important determinants of the outcome of VEE infection and reservoir potential of these rodents.

## Abstract 2

VEEV killed 2 allopatric populations of cotton rats but not a sympatric population from Florida.

Vertebrate reservoir hosts play an important role in maintenance and dissemination of zoonotic pathogens. For arthropodborne viruses (arboviruses), infected hosts generally show little or no disease, which presumably reflects long-term selection for host resistance and possibly virus attenuation (*1*,*2*). Understanding how pathogens affect their reservoir hosts as well as how the reservoir affects fitness and replication of the pathogen could enable better prediction of emergence, reemergence, or extinction of zoonotic pathogens in response to environmental changes. For example, changes in a reservoir host’s habitats and ecology, due to anthropogenic or natural causes, may affect pathogen transmission to humans and domestic animals. A better understanding of reservoir biology and of pathogen-reservoir interactions, particularly their mechanisms of disease resistance, could also facilitate the development of treatment and control strategies for humans and domestic animals (*3*,*4*).

*Venezuelan equine encephalitis virus* (VEEV), a member of the family *Togaviridae*, genus *Alphavirus* (*5*), was first isolated and characterized in 1938 (*6*,*7*) and affects humans and equids in the Americas (*8*,*9*). VEEV strains are classified into 2 epidemiologic groups: enzootic and epizootic. Enzootic strains (subtype I, varieties D and E, as well as related species in the VEE complex comprising subtypes II–VI) have been regularly isolated in lowland tropical forests and swamps in Florida, Mexico, and Central and South America. These enzootic viruses generally circulate between *Culex (Melanoconion)* spp. mosquito vectors and rodent reservoirs and are usually avirulent for and incapable of amplification in equids (*8*). In contrast, epizootic (and epidemic) VEEV strains (subtype I, variety AB and variety C), have been responsible for all major equine outbreaks that have involved other mosquito vectors, primarily *Aedes* and *Psorophora* spp. Epizootics and epidemics have occurred from southern North America to northern South America, and the VEEV strains involved have caused debilitating neurologic disease with high fatality rates in equids (*9*–*11*). In humans, who are tangential hosts during endemic and epizootic cycles, severe febrile illness can become life threatening. Although <1% of infected humans die, permanent neurologic sequelae can occur in survivors, particularly young children (*8*,*12*,*13*).

Serosurveys have found VEEV antibodies in many species of small mammals (*14*–*18*). However, spiny rats (*Proechimys* spp., family Echimyidae*)* and cotton rats (*Sigmodon* spp., family Cricetidae) have been most often implicated as principal reservoir hosts for enzootic strains, based on seroprevalence and experimental infections demonstrating viremia adequate in titer and duration to infect enzootic mosquito vectors (*19*–*22*). Their geographic distributions are different, but overlapping: *Proechimys* spp. are found mainly in Panama, northern Peru, Bolivia, Paraguay, and Brazil; *Sigmodon* spp. are found mainly from southern North America to northern parts of Venezuela, Colombia, and Peru (*23*).

Studies supporting the role of cotton rats as reservoir hosts for enzootic VEEV have investigated viremia and antibody responses as well as horizontal transmission in laboratory settings (*19*–*21*,*24**,**25*). Howard et al. reported illness and death in cotton rats after infection with a Texas epizootic subtype IB strain (*21*). The cause of death was linked more to experimental manipulation than to virus infection. Another study that examined clinical and histopathologic manifestations after infection of rats with Everglades virus (EVEV; subtype II in the VEE complex) reported that although viremia developed and the virus replicated in a wide variety of organs, only 2% died (*25*).

The southern United States has 12 native subspecies of cotton rats (*26*), which differ by as much as 5% in their cytochrome b DNA sequence (*27*). To determine whether responses to infection vary among these geographic populations, we studied infection with 2 different subtypes of enzootic VEEV in 3 populations of cotton rats.

## Materials and Methods

### Animals

Three subspecies of *Sigmodon hispidus* (cotton rat) were used in this study: the Harlan colony, the Texas population, and the Florida population. The Harlan colony consisted of 6- to 8-week-old female rats purchased from Harlan (Indianapolis, IN, USA) from an established colony. Because the exact geographic origin was unknown, cytochrome B mitochondrial gene sequences were amplified by PCR and compared phylogenetically with those from cotton rats from various locations in North America (*27*). The sequences from the Harlan colony grouped with those of animals collected in east Texas, Louisiana, and Tennessee but were outside of the clade from southern Florida (data not shown), which indicated that these rats originated from a nonenzootic region. (Florida is the only VEE complex alphavirus–enzootic region in the United States, aside from the Rocky Mountains, which are outside of *Sigmodon* distribution [*8,23*]). The Texas population consisted of 4- to 12-month-old wild-caught male and female *S. hispidus berlandieri* trapped in Galveston Island State Park, Texas (29.27°N, 94.83°W) (*25*). The Florida population consisted of 3- to 21-week-old male and female cotton rats (*S. hispidus spadicipygus)* trapped in southern Florida (*25*) and used to rear F1 rats for experimental studies. Before inoculation, all rats were tested for antibodies against VEEV, EVEV, and Eastern equine encephalitis virus (EEEV) and acclimated for 3 days in a Biosafety Level 3 animal facility. All experiments included >2 animals as negative controls. All studies were approved by the University of Texas Medical Branch Animal Care and Use Committee.

### Viruses and Infections

VEEV strain Co97-0054 (enzootic ID subtype), isolated in Colombia in 1997 from a sentinel hamster, and strain 68U201 (enzootic IE), isolated in Guatemala from a sentinel hamster and derived from a cDNA clone (*28*), were used for experimental infections. These strains were selected because they had low passage histories and represent the 2 major enzootic VEEV subtypes; strain Co97-0054 has also been used for experimental infection of spiny rats (*22*). Cotton rats were inoculated subcutaneously in the left footpad or left thigh with 3–4 log_10_ PFU of virus, a dose consistent with the maximum amount of VEEV in mosquito saliva (*29*). To determine whether an increase in virus inoculum could change the outcome of the disease, 4 Florida rats were also inoculated with 5–6 log_10_ PFU of VEEV strain 68U201. All animals were observed for signs of illness once a day for 15 days.

To determine the neurovirulence of VEEV in the Florida population, 3 rats (2 months of age) were inoculated intracranially with 3 log_10_ PFU/mL of strain 68U201. Blood samples were collected 24 h after inoculation, and the rats were observed for signs of disease.

### Viremia Assays

Blood samples were collected from the retroorbital sinus for <10 days after inoculation. Serum samples were diluted 1:10 in Eagle minimal essential medium supplemented with 20% fetal bovine serum, gentamicin, and L-glutamine and stored at –80°C. Viremia titers were determined by plaque assay with Vero cells (*30*).

### Antibody Assays

Plaque reduction (80%) neutralization (PRNT) and hemagglutination inhibition (HI) tests were performed (*30*). To detect specific VEEV immunoglobulin M (IgM) antibodies, an IgM-capture ELISA was performed (*31*). Briefly, microplates were coated with anti-rat IgM, diluted 1:500 in carbonate-bicarbonate buffer pH 9.6 (Kirkegaard and Perry Laboratories, Gaithersburg, MD, USA), and incubated at 4ºC for at least 16 h. Subsequently, the following were sequentially added: test serum, mouse immune ascitic fluid prepared against VEEV antigens, anti-mouse conjugate (Kirkegaard and Perry), and ABTS (2,2′-azino-bis[3-ethylbenzthiazoline-6-sulphonic acid])–peroxidase substrate (Kirkegaard and Perry). Test serum samples were diluted at 1:40 in 0.5% bovine serum albumin in phosphate-buffered saline at pH 7.4, and the plates were read by using a spectrophotometer with a 405-nm wavelength filter. The cut-off value was calculated as the mean optical density (OD)_405 nm_ of negative control samples plus 3 standard deviations, or 0.200. Linear regression, the Student *t* test, and analysis of variance were used to analyze data.

## Results

### Clinical Responses and Survival

**Figure 1:**
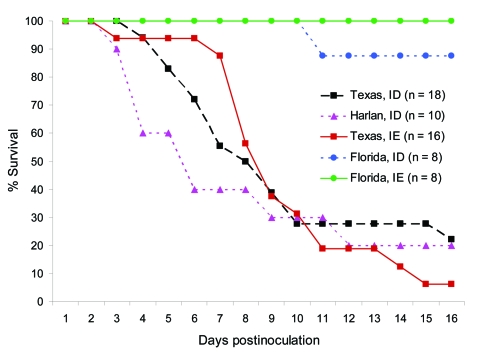
Survival of cotton rats from Florida, Texas, and Harlan after subcutaneous inoculation with 3 log_10_ PFU of enzootic Venezuelan equine encephalitis virus (subtypes IE and ID).

Inoculation of the Florida rats with 3 log_10_ PFU of VEEV strain 68U201 (IE) or Co97-0054 (ID) resulted in no detectable signs of illness and survival rates of 100% and 87.5%, respectively (Figure 1). One Florida rat inoculated with strain Co97-0054 died on day 10 postinoculation without exhibiting any signs of illness. These findings contrasted with the results of VEEV infections of the Texas and Harlan populations. Although the Harlan rats were inoculated with only the subtype ID strain, signs of severe illness developed in all of the Harlan and Texas rats beginning on day 5. Signs included ruffled coats, lack of grooming, lethargy, and for many, signs of encephalitis (incoordination and instability when walking and erratic movements of the head and limbs), dehydration (measured by lack of skin turgor), and anorexia. Most animals that died before day 5 postinoculation showed no prior signs of illness. The average survival time for the Texas population was 6.8 days after inoculation with the subtype ID strain and 8.2 days with the IE strain; for the Harlan colony, it was 5 days after inoculation with the subtype ID VEEV. None of the animals that survived past day 15 died. The 2 sham-inoculated and the 2 noninoculated rats survived without signs of disease.

### Neurovirulence in Florida Cotton Rats

**Figure 2:**
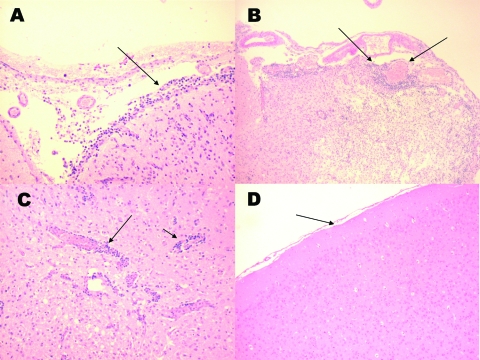
Histologic staining (hematoxylin and eosin) of Florida cotton rat tissues 9 days after intracranial inoculation with 3 log_10_ PFU of enzootic Venezuelan equine encephalitis virus (subtype IE). A) Inflammation of the meninges (arrows). B) Inflammation of the meninges and dilated blood vessels (arrows). C) Perivascular cuffing of blood vessels (arrow). D) Brain from a noninfected rat. (Magnification ×40.)

To determine whether the absence of disease in the Florida population was due to the inability of the virus to penetrate the central nervous system, 3 rats were inoculated intracranially with 3 log_10_ PFU/mL of subtype IE VEEV. Viremia titers at 24 h postinoculation were 6.3, 6.2, and 6.8 log_10_ PFU/mL (mean 6.5). By day 3 postinoculation, all of these rats started showing signs of illness, including ruffling of the fur and lack of movement. By day 9 postinoculation, 1 rat was dead and the other 2 exhibited instability and difficulty in walking, uncoordinated and erratic movements of the head and limbs, dehydration, and anorexia; these animals were euthanized because of the severity of disease. Histopathologic examination of the brains showed clear signs of encephalitis, focal meningoencephalitis (Figure 2, panels A, B) and associated perivascular mononuclear cell infiltration (Figure 2, panel C), and neurophagia, which led to the conclusion that the cause of death was from the viral infection and not from the injection or manipulation of the animals.

### Dose Dependence

To determine whether the Florida population’s apparent resistance to VEE after peripheral infection was dose-dependent, 8 additional animals were inoculated with 5 or 6 log_10_ PFU (4 animals per dose). In each population, 3 (75%) of the rats survived infection (data not shown). One rat inoculated with 6 log_10_ PFU of virus succumbed to disease on day 3, whereas another animal inoculated with 5 log_10_ PFU of virus died on day 11, which suggests that the dose could have affected disease progression. None of the other inoculated animals showed any clinical signs of illness.

### Viremia Titers

**Figure 3:**
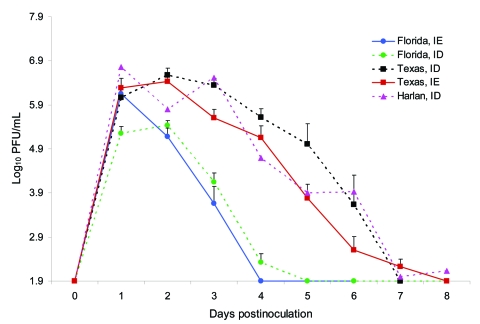
Mean viremia titers of cotton rats from Florida, Texas, and Harlan after subcutaneous inoculation with 3 log_10_ PFU of enzootic Venezuelan equine encephalitis virus (subtypes IE and ID). Bars indicate standard errors of the means.

Viremia profiles for the Florida rats were similar after inoculation (3 log_10_ PFU) with either subtype ID or IE VEEV (p>0.05). Peak viremia titers of 6.2 and 5.4 log_10_ PFU/mL, respectively, occurred at 24–48 h postinoculation, then became undetectable by days 4–5 postinoculation (Figure 3). The Texas population inoculated with 3–4 log_10_ PFU of subtype IE virus and 3 log_10_ PFU of subtype ID VEEV had similar viremia profiles, with peak titers of 6.1 and 6.6 log_10_ PFU/mL, respectively, at 24–48 h.

**Figure 4:**
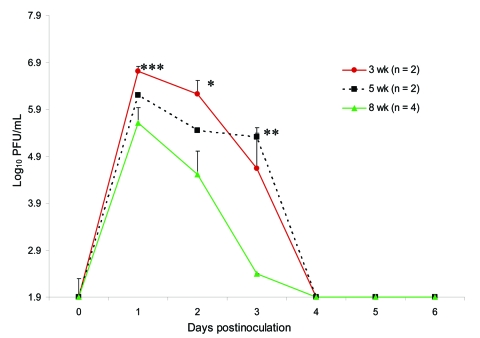
Age-dependent viremia in Florida cotton rats inoculated subcutaneously with 3 log_10_ PFU of subtype IE Venezuelan equine encephalitis virus. Randomly picked female and male animals aged 3–8 weeks were inoculated subcutaneously with 3 log_10_ PFU. Significant differences were detected on day 2 postinoculation (*p = 0.007) and day 3 (**p = 0.02) but not on day 1 (***p = 0.06). Bars indicate standard errors of the means.

We found in the Florida population a correlation between age and peak viremia titers. Younger animals inoculated with the IE virus had higher peak titers on days 1 and 2 postinoculation than did older animals (Figure 4, Table 1). In addition, we observed a significant difference in mean viremia titers on day 2 between 3- and 8-week-old animals and on day 3 between 5- and 8-week-old animals and between 3- and 8-week-old animals (p<0.05).

**Table 1:** Mean viremia titers of Florida cotton rats inoculated subcutaneously with Venezuelan equine encephalitis virus, subtype IE*

Day postinfection	Age, wk		
	3 (n = 2)	5 (n = 2)	8 (n = 4)
1	6.7	6.2	5.6
2	6.2	5.4	4.5
3	4.6	5.3	2.4
4	1.9	1.9	1.9
5	1.9	1.9	1.9
6	1.9	1.9	1.9

*Titers are expressed as log_10_ PFU/mL.; 1.9 log_10_ PFU/mL is the limit of detection of the plaque assay.

### Differences in Virus Titers among Cotton Rat Populations

No differences in viremia profile were observed between the Texas and Harlan rats, for which VEEV infection with subtype ID was generally fatal (p>0.05). Viremia in the Texas and Harlan rats peaked between 24 and 48 h postinoculation, with mean titers of ≈6.7 log_10_ PFU/mL, and was undetectable by day 8 postinoculation. In contrast, significant differences were observed in peak viremia titers between the Florida and Texas rats inoculated with either the ID or IE VEEV subtype (p<0.05). In all cases, Texas rats produced higher titers (24 h postinoculation) and had a longer duration of viremia than the Florida rats, in which no disease was apparent (Figure 3, Tables 2, 3). Similar results occurred when Florida and Harlan rats inoculated with subtype ID were compared.

**Table 2:** Mean viremia titers of Florida, Texas, and Harlan cotton rats inoculated subcutaneously with Venezuelan equine encephalitis virus, subtype ID or IE*

Day postinfection	Cotton rat population and virus subtype					
	Florida, IE	Florida, ID	Texas, IE	Texas, ID	Harlan, ID
1	6.1 ± 0.2	5.2 ± 0.2	6.3 ± 0.2	6.1 ± 0.2	6.8 ± 0.9
2	5.2 ± 0.2	5.4 ± 0.1	6.4 ± 0.1	6.6 ± 0.2	5.8 ± 0.9
3	3.7 ± 0.4	4.1 ± 0.2	5.6 ± 0.2	6.3 ± 0.2	6.5 ± 0.5
4	<1.9	2.3 ± 0.2	3.8 ± 0.3	5.6 ± 0.2	4.7 ± 0.8
5	<1.9	<1.9	2.6 ± 0.3	5.0 ± 0.5	3.9 ± 0.1
6	<1.9	<1.9	2.2 ± 0.2	3.6 ± 0.7	3.9 ± 0.2
7	<1.9	<1.9	<1.9	<1.9	2.0 ± 0.0
8	<1.9	<1.9	<1.9	<1.9	2.1 ± 0.3

*Titers are expressed as log_10_ PFU/mL ± standard error; 1.9 log_10_ PFU/mL is the limit of detection of the plaque assay.

**Table 3:** Statistical comparisons (p values) among viremia titers in Texas and Florida cotton rats inoculated with Venezuelan equine encephalitis virus, subtype IE or ID*

Day postinfection	Texas vs. Florida, subtype IE	Texas vs. Florida, subtype ID	Subtype IE vs. ID, Texas	Subtype IE vs. ID, Florida
1	0.123	**0.018**	0.590	0.078
2	**5.0 × 10^–4^**	**4.69 × 10^–5^**	0.506	0.078
3	**9.75 × 10^–7^**	**2.72 × 10^–6^**	0.506	0.215
4	**2.82 × 10^–10^**	**3.66 × 10^–8^**	0.348	0.751
5	**1.31 × 10^–5^**	**5.71 × 10^–5^**	0.058	1
6	**0.051**	0.010	0.123	–

*Numbers in **boldface** indicate statistically significant differences (analysis of variance).

### Antibody Responses

**Figure 5:**
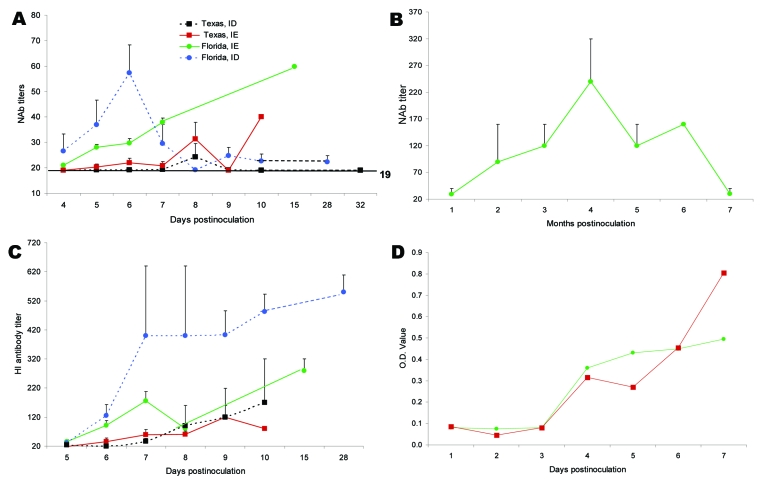
Antibody responses in cotton rats from Florida and Texas. A) Neutralizing antibody (NAb) titers in Florida group (n = 3–11) and Texas group (n = 1–17) inoculated with subtypes IE or ID Venezuelan equine encephalitis virus (VEEV). B) Long-term NAb titers in Florida rats infected with subtype IE VEEV (n = 2). C) Hemagglutination inhibition (HI) antibody titers for Florida (n = 2–10) and Texas (n = 1–16) rats inoculated with subtype IE VEEV. D) Immunoglobulin M antibody titers for Florida and Texas rats infected with subtype IE VEEV (n = 2). OD, optical density.

To determine whether the difference in disease outcome in Florida versus Texas rats was due to a difference in antibody responses, serum was tested by PRNT. In the Texas population inoculated with subtype IE VEEV, low titers of neutralizing antibodies (NAb) were produced by days 5–6 postinoculation (mean titer 20), and mean titers never exceeded 40 by day 10 (Figure 5, panel A); however, detectable NAb were not produced in all animals that died. In contrast, some Florida rats inoculated with the same virus strain had detectable NAb titers by day 4, and all had detectable titers by day 6. NAb titers were significantly lower in the Texas rats on days 5, 6, and 7 (p = 0.02, 0.04, 0.04, respectively).

When Texas rats were inoculated with the subtype ID VEEV strain, even lower NAb titers were produced, despite the development of higher viremia titers during the later stages, compared with those inoculated with subtype IE. Peak NAb titers occurred on day 8 postinoculation (mean 25), and 2 of the surviving animals had no detectable NAb titers (<19) late during infection (day 32). In contrast, in all Florida rats, detectable NAb developed by day 4 and peaked on day 6 (mean 57), when they were significantly higher (p = 0.004).

To determine the duration of the antibody response in the Florida population, we measured NAb titers in 2 rats for 7 months postinoculation. Titers peaked at 4 months and then gradually decreased to the detection limit by 7 months, when the experiment was terminated (Figure 5, panel B).

Because NAb were not detected in all infected animals, serum samples were further tested by HI and IgM ELISA. All rats had detectable HI antibodies by days 5–6 postinoculation. Although the titers were relatively low (<200) on day 6, titers increased constantly over the 10 days tested; no differences in titers were noted between VEEV subtype ID or IE infections in the Texas population. As was found by PRNT, HI antibody titers were higher for the Florida population than the Texas population, and titers were higher in animals inoculated with the ID than with the IE VEEV strain (Figure 5, panel C).

Because of volume limitations of daily blood collection, IgM titrations were performed only on blood samples from euthanized rats (2 rats per day per group). Both Florida and Texas populations had similar IgM titers during the first 7 days postinoculation, regardless of the virus used. Mean titers ranged from OD 0.3 on day 4 to OD 0.8 by day 7 in the Texas population and 0.5 in the Florida population (Figure 5, panel D).

In summary, NAb titers were higher in Florida rats inoculated with subtypes ID and IE than in Texas rats inoculated with the same viruses. This finding suggests that these animals may have mounted a more rapid and effective immune response that protected against severe VEE infection.

## Discussion

### Reservoir Status and Potential

*S. hispidus,* a main reservoir host of VEE complex alphaviruses, comprises >22 subspecies in North America alone (*26*) that differ by up to 5% in their cytochrome b mitochondrial DNA sequences (*27*). Because some but not all North American populations occur in regions enzootic for VEE complex alphaviruses (e.g., EVEV in Florida), we attempted to better understand the host-VEEV interactions by inoculating 3 different populations with enzootic VEEV strains. Cotton rats from the enzootic area of southern Florida (sympatric with EVEV) responded to VEEV infection as expected: moderate viremia titers, seroconversion by days 4–5 postinoculation coincident with viremia cessation, and few deaths and little detectable disease. This apparently commensal relationship could reflect long-term selection for cotton rat resistance to EVEV in Florida. Although EVEV is a relatively benign virus in laboratory rodents, the ancestral form of EVEV, believed to be a subtype ID VEEV strain from Panama or South America, is more virulent (*32*).

In contrast, rats from 2 nonenzootic locations (the Harlan and Texas populations) had dramatically different outcomes: severe disease often culminated in clinical signs of encephalitis and high mortality rates. This difference in disease and survival was more pronounced than that reported in other studies of VEEV-reservoir host interactions, some of which suggested that cotton rats die because of experimental manipulations or anesthesia rather than from a viral cause (*19*–*21**,**24*). Our results indicate that VEEV was the cause of death for most or all of our rats, and signs of encephalitis were consistent with those described in VEEV-infected mice or guinea pigs (*8*,*33**,**34*). The rats infected in previous studies had several different geographic origins (Arizona, North Carolina, Florida, and Panama). Of these, only Panama and Florida are enzootic for VEE complex alphaviruses. North Carolina is enzootic for another closely related alphavirus, EEEV, for which birds are thought to be the main reservoirs (*35*). These allopatric rat populations should be reexamined to further test the hypothesis that lack of exposure to VEE complex or other alphaviruses has resulted in no selection for resistance. On the basis of previous susceptibility studies, the peak viremia titers in all infected cotton rats were sufficient to infect known enzootic vector mosquitoes (*36**,**37*).

Reservoir hosts play an important role in the maintenance and spread of zoonotic viruses. They generally show little or no disease after infection with VEEV and most other zoonotic viruses, which presumably reflects long-term selection for resistance (*1*,*2*). This resistance is little studied and poorly understood, yet it might provide insight into improved treatments for arbovirus infections in humans. Our findings may also have implications for VEEV ecology, especially in the event of virus introduction into a nonenzootic region, as occurred during the 1971 Texas VEEV epizootic (*38**,**39*). During such a scenario, virus-induced deaths might deplete cotton rat populations, depending on the VEEV transmission levels and the length of the outbreak.

### Viremia and Immunologic Responses

The differences in viremia profiles exhibited by the VEE-sympatric versus VEE-allopatric cotton rat populations could explain the different disease outcomes. Although peaks of viremia titers were similar for both subspecies, durations of viremia differed. The prolonged viremia observed in the Texas and Harlan animals may reflect a poor or delayed innate or adaptive immune response, which led to sustained viral replication and death. This could be an indirect effect of replication in lymphatic tissues leading to immunosuppression. Although peak viremia titers appeared to be age-dependent in the Florida population inoculated with the subtype IE VEEV strain, disease outcomes between age groups, which might reflect maturation of the immune system, did not differ significantly. Antibody titers in both populations of cotton rats after inoculation with either virus strain were relatively low.

These findings contrast with results of a previously published study of EVEV infection of cotton rats from the same 2 geographic regions (*25*). In that study, both subspecies of rats survived infection, exhibited similar peak viremia titers, and had high antibody titers within 9 days postinoculation; it was suggested that the innate immune response was involved. EVEV, enzootic in South Florida, is less virulent in laboratory rodents than in most other viruses in the VEE complex, including the subtypes we used (*19*,*40*). Presumably due to this lack of virulence, Florida and Texas strains of cotton rats tested produced protective antibodies and survived EVEV infection (*25*)*.*

Our data from the same 2 rat populations but inoculated with more virulent stains of VEEV present a different picture. Although the innate immune response may be involved as well, antibody detection correlated with the disappearance of viremia. The capability of Florida cotton rats to produce antibodies against VEEV early may allow them to better control replication and survive. Antibodies against VEEV persist for at least 6 months in laboratory-infected cotton rats (*25*). The average lifespan of cotton rats in nature is estimated to be ≈6–8 months (*41*).

To identify protective mechanisms in the Florida population, additional studies focusing more on the innate immune responses of enzootic and nonenzootic cotton rat populations are needed. This could be approached by cross-breeding the Texas and the Florida rats and studying the offspring. Elucidation of protective mechanisms may be useful for developing new strategies for treating human or equine infections.
